# Molecular Advances in the Treatment of Advanced Gastrointestinal Stromal Tumor

**DOI:** 10.1093/oncolo/oyad167

**Published:** 2023-06-14

**Authors:** Vinayak Venkataraman, Suzanne George, Gregory M Cote

**Affiliations:** Dana-Farber Cancer Institute, Department of Medical Oncology, Boston, MA, USA; Mass General Hospital Cancer Center, Center for Sarcoma and Connective Tissue Oncology, Boston, MA, USA; Dana-Farber Cancer Institute, Department of Medical Oncology, Boston, MA, USA; Mass General Hospital Cancer Center, Center for Sarcoma and Connective Tissue Oncology, Boston, MA, USA

**Keywords:** sarcomas, cancer diagnostics and molecular pathology, new drug development and clinical pharmacology

## Abstract

Most gastrointestinal stromal tumors (GIST) are driven by activating mutations in Proto-oncogene c-*KIT* (*KIT*) or *PDGFRA* receptor tyrosine kinases (RTK). The emergence of effective therapies targeting these mutations has revolutionized the management of advanced GIST. However, following initiation of first-line imatinib, a tyrosine kinase inhibitor (TKI), nearly all patients will develop resistance within 2 years through the emergence of secondary resistance mutations in *KIT*, typically in the Adenosine Triphosphate (ATP)-binding site or activation loop of the kinase domain. Moreover, some patients have de novo resistance to imatinib, such as those with mutations in *PDGFRA* exon 18 or those without *KIT* or *PDGFRA* mutation. To target resistance, research efforts are primarily focused on developing next-generation inhibitors of KIT and/or PDGFRA, which can inhibit alternate receptor conformations or unique mutations, and compounds that impact complimentary pathogenic processes or epigenetic events. Here, we review the literature on the medical management of high-risk localized and advanced GIST and provide an update on clinical trial approaches to this disease.

## Teaser

This article reviews the literature on the medical management of high-risk localized and advanced GIST and provides an update on clinical trial approaches to this disease.

**Implications for Practice**

The management of advanced GIST has profoundly changed over the past 2 decades. This review highlights improvements in the understanding of molecular pathogenesis of both primary and TKI resistant GIST and the sequential development of effective targeted therapies. It emphasizes the importance of upfront molecular testing to personalize treatment and optimize outcomes. It summarizes clinical trial results to provide an evidence base for current practice and highlights unique drug associated toxicities. It concludes with summary of the pipeline of novel agents being evaluated in this disease.

## Background

Gastrointestinal stromal tumors (GIST), the most common soft-tissue sarcoma of the gastrointestinal (GI) tract, are diagnosed in over 6000 patients each year in US.^1^ Globally, 10-15 per million people are diagnosed each year with the highest incidence in China, Taiwan, Korea, and Norway.^2^ GIST have the highest incidence in adults over 60,^1,2^ but certain familial mutations can predispose younger patients.^3-5^

GIST originate most commonly in the stomach (55%-60%) or duodenum (30%-35%).^2,6^ They can be discovered incidentally or due to abdominal symptoms or bleeding.^6,7^ Metastatic lesions are typically found in the liver or peritoneum.^8^ Multiple imaging modalities can identify GIST; endoscopic ultrasound with fine needle aspiration biopsy is the preferred diagnostic modality.^9,10^

For patients with fully resectable GIST, the mainstay of treatment is complete surgical resection with en bloc tumor removal and negative margins.^6,11-14^ Traditional chemotherapy is ineffective, and radiation is typically reserved for palliation or in clinical scenarios where patients are not candidates for surgery or medical therapy.^11,12,15^

For resectable tumors, the recurrence risk after definitive treatment is based on several prognostic factors: tumor size, location (gastric vs. nongastric), tumor rupture, and mitotic index.^16-23^Table 1 highlights criteria for risk stratification, based on size (in cm), mitotic rate (in 5 mm^2^), and location.^16^

**Table 1.** Risk of progressive disease based on mitotic rate, size, and location.^16^

Tumor parameters		Risk of progressive disease (%)			
Mitotic rate (per 5mm)	Size (cm)	Gastric	Duodenum	Jejunum/ileum	Rectum
≤5	≤2	None (0%)	None (0%)	None (0%)	None (0%)
	2 to ≤5	Very low (1.9%)	Low (8.3%)	Low (4.3%)	Low (8.5%)
	5 to ≤10	Low (3.6%)		Mod (24%)	
	>10	Mod (10%)	High (34%)	High (52%)	High (57%)
>5	≤2	None		High	High (54%)
	2 to ≤5	Mod (16%)	High (50%)	High (73%)	High (52%)
	5 to ≤10	High (55%)		High (85%)	
	>10	High (86%)	High (86%)	High (90%)	High (71%)

TKI therapy directed against identified oncogenic drivers of GIST, such as mutated Proto-oncogene c-*KIT (*KIT*)* and *PDGFRA*, has revolutionized the treatment of advanced GIST; however, treatment response and resistance patterns vary based on molecular factors.^11^

In this review, we discuss the molecular subtypes of GIST, clinical use of TKIs in resectable high-risk GIST or advanced GIST, and review novel approaches to counteract resistance. We will conclude with discussion of future treatment investigations.

## Materials and Methods

A literature search of English language-only references was conducted in PubMed using the search terms GIST, gastrointestinal stromal tumor, highrisk, advanced, and metastatic. In addition, a ClinicalTrials.gov search for clinical trials recruiting patients with GIST was conducted.

## The Many Molecular Subtypes of GIST

GIST are believed to originate from the interstitial cells of Cajal, or a precursor cell, along the GI tract.^17,18^ Morphology, they can be either spindled (70%), epithelioid (20%), or mixed (10%), and typical immunohistochemical markers include CD117 and DOG1.^17-21^

Most GIST (85%) are characterized by activating mutations in one of 2 receptor tyrosine kinases: (a) *KIT* (80%) and (b) platelet-derived growth factor receptor A (*PDGFRA*) (5%).^21-23^

**Figure 1.**
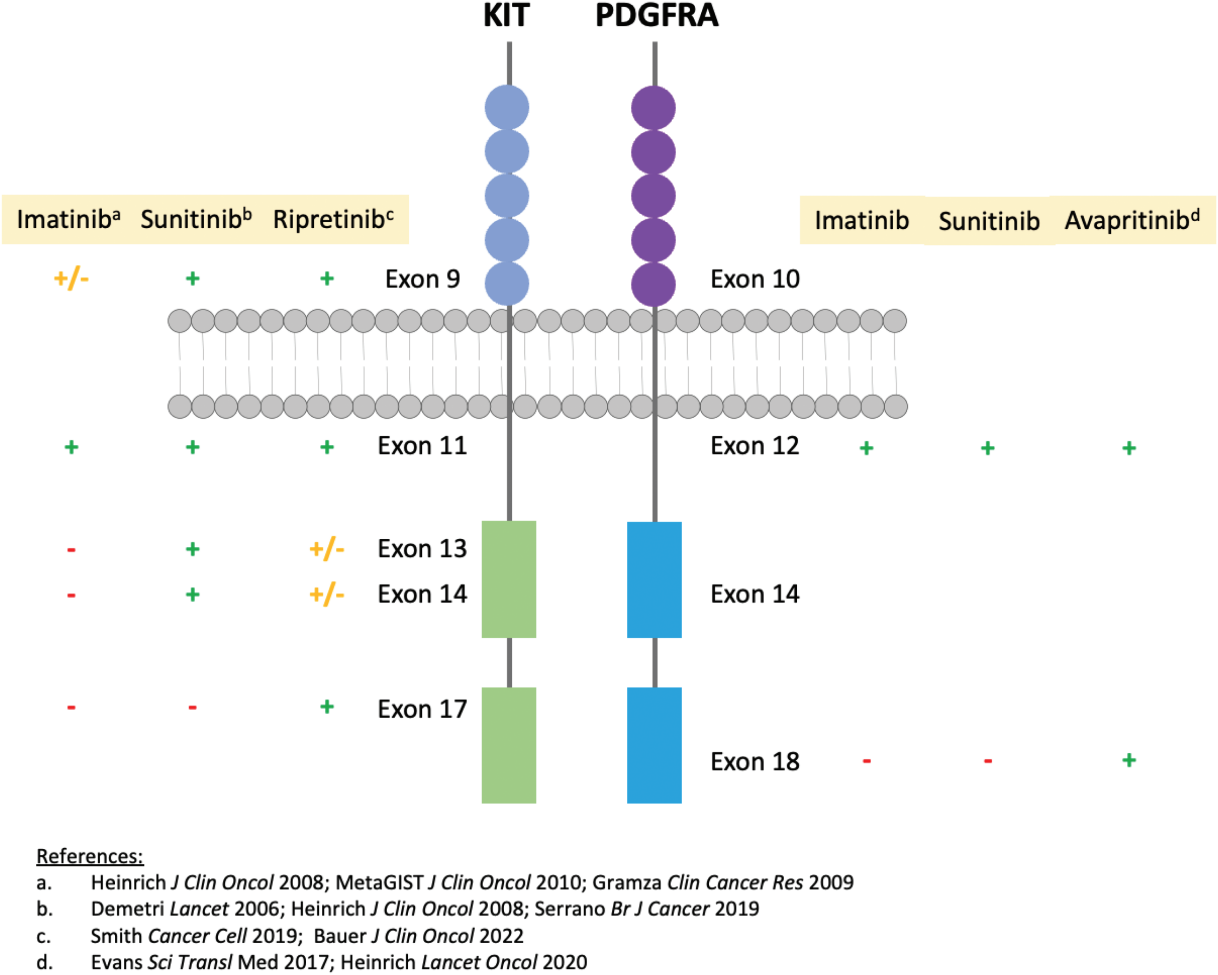
Structure of KIT and PDGFRA with primary and secondary resistance mutations to TKIs noted by exon.^24-33^

The structures of KIT and PDGFRA, shown in Fig. 1, share homology and are comprised of an extracellular, a transmembrane, a juxta-membrane, and a kinase domain, the latter including an Adenosine Triphosphate (ATP)-binding domain and activation loop. In normal cellular function, both receptors dimerize in a ligand-dependent manner and activate downstream pathways, such as mitogen-activated protein kinase and mammalian target of rapamycin (mTOR) pathways. In GIST, activating *KIT* and *PDGFRA* mutations lead to ligand-independent dimerization, constitutive activation, and subsequent uncontrolled intracellular signaling and cell growth.^21-23^

De novo activating *KIT* mutations typically occur in one of 4 exons: exon 11 (67% of all GIST, including non-*KIT*-mutated GIST), exon 9 (10%), 13 (1%), and 17 (1%).^21-23^

Exon 11 mutations modify the juxta-membrane domain’s structure, interfering with auto-inhibitory processes. Exon 9 (ie, AY502-503 insertion) mutations modify the extracellular domain’s structure, making it resemble a ligand-activated conformation. Exon 13 (ie, K642E) and exon 17 (ie, D820Y, N822K, and Y823D) mutations modify the kinase domain’s ATP-binding domain and activation loop, respectively, and in the case of exon 17, stabilize an active conformation.^21-23^

*PDGFRA* mutations occur predominantly in exon 18 (90%), which encodes the activation loop. Exon 18 mutations are further classified as D842V (62%) or non-D842V (28%). Exon 12 (ie, V561D) and exon 14 (ie, N659K) mutations, which affect the juxta-membrane and ATP-binding domains, respectively, are less common. *PDGFRA* mutant GIST have strong predilection for the stomach and lower metastatic potential than *KIT*-mutant GIST.^21-23,34-36^

The remaining 15% do not harbor *KIT* or *PDGFRA* mutations and were previously characterized as “wildtype” GIST. These patients are now known to encompass several molecular subtypes.^21-23^

Patients with Succinate dehydrogenase (SDH)-deficient GIST have either a loss-of-function mutation in a subunit of the SDH complex, or epigenetic silencing typically of SDH-C^3,4,21-23^ Alterations can be either germline or somatic. Patients are often younger and may have multifocal gastric disease, lymph node involvement, and a relatively indolent course. SDH-deficient GIST is also a component of Carney’s triad, alongside extra-adrenal paragangliomas, and pulmonary chondromas.^5,37-39^

Additional subtypes include NF-1 related GIST, which may be associated with neurofibromatosis type 1, also typically indolent,^21,22,37^ as well as *BRAFV600E*^40,41^ and NTRK-fusion GIST.^42^

## The Emergence and Advancements of TKI Therapy

Novel TKIs, developed to inhibit aberrant *KIT* and *PDGFRA* mutations, have sequentially been approved for treatment of advanced GIST. Differential response to TKIs based on primary and secondary mutations highlights the potential importance of mutation testing with treatment initiation and at times of progression.

Imatinib, a relatively selective oral kinase inhibitor, approved for its BCR-ABL inhibition in chronic myelogenous leukemia,^43^ also exhibited inhibitory potential against mutated forms of KIT and PDGFRA. It competitively inhibits ATP from binding the ATP-binding domain and stabilizes the inactive conformation. Imatinib was found effective in 2 international phase III trials^44,45^ and Food and Drug Administration (FDA) approved in 2001 as the first treatment for patients with advanced GIST.^46^

After the approval of imatinib, subsequent research showed differential responses based on molecular subtype. Imatinib was found to be most active in *KIT* exon 11 mutations.^24,47^*KIT* exon 9 mutations, however, confer some degree of primary resistance, which may be overcome with higher doses.^25^ PDGFRA D842V mutations, and those without *KIT* or *PDGFRA* driver mutations, showed no response.^34,47^

Despite initial efficacy, most patients develop resistance to imatinib within 2 years. This is typically due to secondary *KIT* mutations within the kinase domain by either interfering with drug binding within the ATP-binding domain (exon 13/14 mutations) or b modifying the activation loop to stabilize the active conformation (exon 17/18 mutations).^23,26,48-51^Figure 1 shows the current understanding of primary and secondary resistance to select TKIs in *KIT* or *PDGFRA* mutant GIST.

Additional oral multitargeted TKIs were approved in sequential fashion in attempt to overcome secondary resistance.

Sunitinib and regorafenib work similarly to imatinib; however, they have a broader spectrum of activity that includes anti-angiogenic inhibition of vascular endothelial growth factor receptors 1-3 (VEGFR1-3). Sunitinib delayed time to tumor progression (TTP) compared to placebo (27 vs. 6 weeks) in phase III testing after imatinib failure^27,52,53^ and was FDA approved in 2006 to treat imatinib-refractory or intolerant patients.^54^ Regorafenib improved progression-free survival (PFS) compared to placebo (4.8 vs. 0.9 months) and was FDA approved in 2013 for patients after sunitinib failure.^55,56^

Both sunitinib and regorafenib have shown some efficacy against secondary *KIT* mutations. In the clinical setting, sunitinib was found to have activity against ATP-binding domain mutations (exon 13/14), but not activation loop mutations (exon 17/18).^28^ In the preclinical setting, regorafenib has activity against exon 17 mutations with less impact on exon 13 mutations.^29^

Ripretinib is a switch-control targeting agent which inhibits KIT and PDGFRA by stabilizing their inactive conformation. Preclinical models demonstrate a wide spectrum of activity against several *KIT* mutations, including within the kinase domain.^30^ Ripretinib significantly improved PFS compared to placebo (7.0 vs. 1.0 months) in the fourth line and beyond setting,^57,58^ and was FDA approved in 2020.^59^

*PDGFRA* D842V mutations on exon 18, which occur in roughly two-thirds of *PDGFRA* mutant GIST, showed no response to any approved TKIs.^34,47^ Early-generation TKIs competitively inhibit the ATP pocket, thus stabilizing the inactive conformation; however, the D842V mutation shifts the PDGFRA kinase protein into the activated conformation, which renders the ATP pocket unavailable.^31,60,61^

Avapritinib, an oral, selective KIT and PDGFRA inhibitor, readily fits into the ATP binding pocket, with the activation loop in an open, aspartate (D)-phenylalanine (F)-glycine (G) motif (DFG)-in conformation^23,31,60^ In a phase I study, avapritinib was found to be active in patients with *PDGFRA* D842V mutant GIST with an ORR of 88%.^32,62^ This study led to FDA and European Medicines Agency approvals in 2020 use in GIST with *PDGFRA* exon 18 and with *PDGFRA* D842V mutations, respectively.^57,58^

The 15% of patients with GIST that do not harbor a mutation in either *KIT or PDGFRA* have fewer treatment options. This GIST subtype does not respond to imatinib,^24,25,47^ and has some, but limited, response to alternate TKIs, such as sunitinib.^63,64^ Patients with SDH-deficient GIST and NF-related GIST are generally referred for clinical trial participation. Patients whose GIST harbors *BRAF* or *NTRK* alterations may benefit from BRAF/MEK^40,41^ and NTRK^42^ inhibition, respectively.

## TKI Use in High-Risk Resectable GIST

The primary treatment of resectable GIST remains surgery^11,13,14^; however, certain features portend a high recurrence risk.^65^ Postoperative imatinib therapy demonstrated benefit in 3 international phase III studies (ACOSOG Z9001, EORTC 62024, SSG XVIII).^66-70^

SSG XVIII was a 400-patient, open-label phase III trial with high-risk GIST with randomization to receive postoperative imatinib for 1 year or 3 years. After a median follow-up of 54 months, the 5-year recurrence-free survival (RFS) and overall survival (OS) were significantly greater in the 3-year arm compared to 1-year arm (66% vs. 48%, HR 0.46, *P* < .001 for RFS; 92% vs. 82%, HR 0.45, *P* = .02 for OS).^69^ Patients with *KIT* exon 11 mutant GIST derived the greatest benefit (RFS 71% vs. 41%).^70^ Additional studies, one phase II (PERSIST-5)^71^ and one single-center retrospective,^72^ have demonstrated potential benefit of continuing postoperative imatinib for 5 or more years.

The optimal length of postoperative imatinib is unclear. For patients tolerating therapy, in some countries, it is not uncommon to continue postoperative imatinib beyond 3 years. This is especially the case with evidence that imatinib is not cytotoxic, but rather suppresses GIST growth, and most recurrences in adjuvant trials occurred within a year of discontinuing postoperative imatinib.^69,72-74^

Molecular testing has emerged as a critical tool to optimize decision making for adjuvant treatment in high-risk patients. For example, patients without *KIT* or *PDGFRA* mutant GIST, and those with *PDGFRA D842V* mutations do not benefit from postoperative imatinib.^69,70^

## Management of Advanced GIST

### Imatinib for the Majority

Imatinib remains the standard-of-care frontline treatment for most patients with advanced GIST.

One phase III study evaluated standard-dose (400 mg daily) compared with highdose (800 mg daily) in 746 patients. After a median follow-up of 4.5 years, the median PFS and OS did not differ between groups (18 vs. 20 months for PFS, and 55 and 51 months for OS, respectively).^45^

Imatinib was well tolerated in both groups. Common toxicities included fatigue, diarrhea, rash, mild cytopenias, and fluid retention. Patients had higher rates of grade 3+ toxicities with 800 mg imatinib (63%) compared with 400 mg imatinib (43%). Dose reductions and delays were more common with 800 mg imatinib (58% and 59%, respectively) than 400 mg imatinib (16% and 38%).^45^

Patients who crossed over from 400 to 800 mg imatinib at progression saw some effect, with 3% achieving partial response (PR) and 28% stable disease (SD), and a postcrossover median PFS of 5 months. This benefit in dose escalation, however, was limited to primary *KIT* exon 9 mutant tumors.^45^ A separate phase III trial (EORTC-ISG-AGITG) of 946 patients, also randomized to imatinib 400 and 800 mg arms, had similar results upon crossover with 2% achieving PR and 27% achieving SD, and postcrossover median PFS of 3 months.^75^

Post hoc analyses revealed patients with *KIT* exon 11 mutation had better response to imatinib therapy compared to those with *KIT* exon 9.^24,47^ A meta-analysis of both phase III trials showed that patients with *KIT* exon 9 mutation who received high-dose imatinib had 42% higher median PFS and numerically, but not significantly better OS.^25^ Patients with *PDGFRA* D842V and without *KIT* or *PDGFRA* mutant GIST were confirmed imatinib nonresponders.^24,25,34,47^

In summary, imatinib remains first-line treatment for most patients with GIST. The standard-dose (400 mg/day) is appropriate for patients with *KIT* exon 11 mutant tumors; however, patients with *KIT* exon 9 mutated GIST may benefit from a higher starting dose (800 mg/day). A small portion of patients with *KIT* exon 9 mutation who progress on 400 mg may respond to escalation. Patients with *PDGFRA* D842V mutant GIST or without *KIT* or *PDGFRA* mutant GIST should not receive imatinib.

### Avapritinib for PDGFRA Exon 18 Mutation

As discussed above, patients with *PDGFRA* exon 18 D842V mutations do not respond to imatinib.^76,77^

Avapritinib was initially evaluated in NAVIGATOR, an open-label phase I trial of 250 patients with unresectable GIST, 56 of whom harbored a *PDGFRA* exon 18 D842V mutation.^32,62^ In the initial report, 49 D842V patients (88%) had an objective response with 5 patients (13%) achieving a CR. At a median follow-up of 15.9 months, the estimated 1-year and 2-year OS were 91% and 81%, respectively.^32^ A subsequent analysis, performed at median follow-up of 27.5 months, showed similar ORR of 91% with median duration of response of 28 months, median PFS of 34 months, and median OS not yet reached.^62^

Avapritinib had similar toxicities to other TKIs (fatigue, diarrhea, peripheral edema, cytopenias); however, it produced notable safety signals for cognitive effects (eg, impairment, mood changes, hallucinations, disordered sleep) and for intracranial hemorrhage. Among participations, 40% had cognitive effects of any grade, a majority (58%) grade 1, and 2% had intracranial hemorrhage (grade 3, nonfatal). The trial established 300 mg as the recommended dose, though 84% of patients did require at least one dose reduction or hold.^32^ A longer-term analysis reaffirmed these findings with 96% requiring dose modification or delay, 46% of participants developing cognitive effects (any grade), and 3% developing intracranial hemorrhage.^62^

Avapritinib is the first drug approved for a specific molecular subtype of GIST establishing the standard-of-care for patients with PDGFRA D842V, exon 18 mutant GIST.

## Subsequent Lines of Treatment

Several TKIs have been approved for treatment of imatinib-refractory GIST.

### Sunitinib

Sunitinib is FDA-approved for patients who are refractory or intolerant to imatinib therapy.^54^

Sunitinib was evaluated in a randomized, placebo-controlled phase III trial of 312 patients with imatinib-resistant GIST. Due to significant treatment effect at interim analysis, the trial was unblinded early. The median TTP was longer with sunitinib (27.3 weeks) compared with placebo (6.4 weeks), as was median PFS (24 vs. 6 weeks). Sunitinib was generally well-tolerated with infrequent dose reductions (11%) and interruptions (28%).^27^

A treatment-use study followed patients with imatinib-resistant GIST who received sunitinib as part of an expanded access program. In total, 1124 patients were recruited across 34 countries and had median TTP of 8.3 months and median OS of 16.6 months.^52^

Common toxicities (any grade) included fatigue, diarrhea, hand-foot syndrome/skin changes, hypertension, and mild cytopenias.^27,52,53^ In a longitudinal follow-up analysis, some patients (13%) developed clinically significant hypothyroidism, mostly grade 1 to 2 (11%). Adverse cardiac events, such as reduced ejection fraction (EF) and left ventricular (LV) dysfunction, occurred (11%); however, grade 3 or higher events were uncommon (<1%).^52^

Sunitinib was initially studied at dosing of 50 mg in a 4-weeks on/2-weeks off regimen which remains the FDA-approved dose; however, subsequent trials evaluated continuous 37.5 mg daily which suggested similar outcomes and remains a guideline-recommended option.^78^

Molecular analyses demonstrated sunitinib is active against multiple *KIT* mutations. Interestingly, median PFS and OS were significantly longer in *KIT* exon 9 than *KIT* exon 11 (PFS: 19.4 vs. 5.1 months; OS: 26.9 vs. 12.3 months, respectively). Patients with secondary *KIT* exon 13/14 mutations had longer median PFS than patients with secondary *KIT* exon 17/18 (7.8 vs. 2.3 months).^28,29^

Sunitinib is the only second-line therapy approved for molecularly unselected patients with GIST. It is well-tolerated with a manageable side effect profile.

### Regorafenib

Regorafenib is FDA-approved for patients refractory or intolerant to imatinib and sunitinib after demonstrating efficacy in phase II and III studies.^55,56^

GRID was a randomized, placebo-controlled phase III trial of patients with GIST after prior imatinib and sunitinib. Median PFS was 4.8 months in the regorafenib group compared to 0.9 months in the placebo group (HR 0.27, CI 0.19-0.39). No difference was noted in OS (HR 0.77, CI 0.42-1.41); however, 85% of participants in the placebo arm crossed over to receive regorafenib. Regorafenib was dosed at a starting dose of 160 mg daily for 3-weeks on/1-week off and generally well-tolerated. The most common treatment-related grade 3 or higher events were hypertension, hand-foot syndrome, and diarrhea. Dose modifications (72%) were common, but permanent discontinuation due to toxicity was infrequent (6%).^56^

Post hoc molecular analysis demonstrated regorafenib is active against certain *KIT* secondary mutations, notably exon 17/18. Regorafenib has some activity against *KIT* exon 13/14 mutations, with the exception of exon 13 V654A mutation.^29^

Regorafenib is the only TKI approved in the third line for advanced GIST.

### Ripretinib

Ripretinib^30^ has been studied in 2 phase III trials,^33,79^ and is FDA-approved for treatment in the fourth-line setting.^59^

INVICTUS was a 129-patient, double-blind, placebo-controlled phase III trial in advanced, multidrug refractory GIST. After a median follow-up of 6.3 months, the median PFS was greater for ripretinib (6.9 months) than for placebo (1 month) (HR 0.15, CI 0.09-0.25). Sixty-six percent of patients assigned to the placebo arm crossed over to ripretinib upon progression. The median OS was significantly higher for ripretinib (15 months) compared to placebo (6.6 months) (HR 0.36, CI 0.21-0.62).^79^

The starting dose of ripretinib is 150 mg daily. The phase I study of ripretinib allowed for dose-escalation to 150 mg BID at disease progression. Of the 142 total participants, 67 participants in the second (*n* = 10), third (*n* = 17), and fourth (*n* = 40) line setting underwent dose-escalation and had postescalation median PFS of 5.6, 3.3, and 4.6 months, respectively.^80^ Additionally, a preplanned analysis of INVICTUS demonstrated that dose-escalation in 43 participants led to a postescalation median PFS of 3.7 months.^81^

Given the benefit seen in INVICTUS, ripretinib was investigated in the second-line setting. The INTRUIGE study was a double-blind, randomized phase III trial of 453 patients comparing ripretinib to sunitinib after imatinib failure. The median PFS did not differ between the ripretinib and sunitinib arms in the total population (8 vs. 8.3 months, respectively) and in the *KIT* exon 11 cohort (8.3 vs. 7.0 months, respectively). However, patients with *KIT* exon 9 mutation had greater benefit with sunitinib over ripretinib (13.8 vs. 5.5 months, HR 2.85, CI 1.48-5.48).^33^

Though ripretinib did not demonstrate PFS benefit in the total population, an exploratory circulating tumor DNA (ctDNA) analysis of INTRUIGE revealed differential PFS based on mutational profile. Namely, patients with ctDNA detection of *KIT* exon 11 and 17/18 mutations (*n* = 52) had significantly improved PFS on ripretinib (*n* = 27) compared to sunitinib (*n* = 25) (14.2 vs. 1.5 months, HR = 0.22, *P* < .001). Alternatively, patients with *KIT* exon 11 and 13/14 mutations (*n* = 41) had shorter PFS on ripretinib (*n* = 21) compared to sunitinib (*n* = 20) (4.0 vs. 15.0 months).^82^

Based on these analyses of INTRIUGE, the National Comprehensive Cancer Network (NCCN) recommends ripretinib as an option for patients intolerant to sunitinib.^83^ Moreover, a randomized phase 3 trial is planned to compare sunitinib versus ripretinib in *KIT* exon 11/17/18-mutant GIST.^84^

Ripretinib is generally well-tolerated with common toxicities including fatigue, nausea, alopecia, hand-foot syndrome, and diarrhea. The most common grade 3+ toxicities included lipase elevation, hypertension, fatigue, and hypophosphatemia.^79^

### Avapritinib

Avapritinib is approved for use in *PGFRA* exon 18 mutated GIST^57^ based on results from the NAVIGATOR trial described above.^32,62^

The VOGAGER phase III study evaluated avapritinib compared to regorafenib in a molecularly unselected treatment-refractory GIST population of patients with *KIT* or *PDGRA* mutant GIST, and avapritinib was not found to be superior to regorafenib.^85^

## Future Direction

Nearly all advanced GIST with *KIT* or *PDGFRA* mutations will eventually develop resistance to currently available TKIs. Developmental therapeutic efforts are focused on strategies to overcome secondary resistance mutations and to identify active treatments for patients without *KIT* or *PDGFRA* mutant GIST.

In addition, the role of circulating tumor DNA (ctDNA) continues to evolve with potential to: monitor for disease relapse, assess treatment response, and identify resistance mutations. Prospective efforts to optimize its role in care and research are ongoing, but with promising initial results.^82,86-91^

### Overcoming KIT Resistance Mutations

Resistance to KIT inhibition is typically due to secondary mutations within the ATP-binding domain (exon 13/14 mutations) or activation loop (exon 17/18 mutations). Most approved TKIs, such as imatinib and sunitinib, are type II inhibitors and block the kinase domain only when in its inactive confirmation; however, certain activation loop mutations, such as *KIT* exon 17 D816V, stabilize the active confirmation. Type I inhibitors hold promise as they can access and inhibit the active conformation.^23,26,48-51^

### Bezuclastinib

Bezuclastinib (CGT9486, previously PLX94986: Cogent Biosciences), is an oral type I KIT inhibitor with high in vitro activity against primary *KIT* exon 9 and 11 mutations, as well as secondary *KIT* exon 17/18 mutations, including exon 17 D816V mutation.^92^

A 2-part phase Ib/IIa trial of bezuclastinib in imatinib-resistant GIST demonstrated single-agent safety and efficacy. Common AEs, mostly grade 1-2, included fatigue, AST elevation, diarrhea, and nausea; grade 3 or higher AEs included anemia and hyperuricemia. Single-agent ORR, CBR, and median PFS at the recommended phase II dose (RP2D) of 1000 mg daily, was 8%, 50%, and 5.75 months, respectively. Combined sunitinib and bezuclastinib at RP2D were confirmed safe, with anticipated adverse events for each agent, and had heightened efficacy with ORR 20%, CBR 80%, and PFS 12.1 months. ctDNA analysis demonstrated single-agent bezuclastinib reduced circulating *KIT* exon 17/18 mutations and combined therapy further suppressed *KIT* exon 13/14 mutations^93^

A phase III randomized open-label trial of CGT9486 and sunitinib versus sunitinib alone (NCT05208047) is currently underway in patients with imatinib resistance or intolerance.^94^

#### IDRX-42

IDRX-42 (Previously M4205: IDRx, Inc.), is a highly selective, type II KIT inhibitor designed to have broad activity against common primary and secondary *KIT* mutations, such as exon 13 V654A. Preclinical testing of IDRX-42 in patient-derived, multidrug-resistant GIST xenograft mice models showed tumor shrinkage and reduction in mitoses.^95^ IDRX-42 is currently in first-in-human phase I testing (NCT05489237).^96^

#### THE-630

THE-630 (Theseus Pharmaceuticals) was also designed as an oral pan-KIT inhibitor with activity against primary and secondary *KIT* mutations. Preclinical testing of THE-630 in GIST demonstrated potent inhibition against both ATP binding domain and activation loop mutations. In ATP binding domain mutations, such as V654A, THE-630 produced greater tumor growth inhibition compared with ripretinib (86% vs. 26%). In activation loop mutations, such as N822K and D820A, THE-630 compared favorably with sunitinib (88% vs. 25%) and ripretinib (59% vs. 1%), respectively.^97^ THE-630 is currently undergoing first-in-human phase I/II dose-escalation and dose-expansion testing (NCT05160168).^98^

#### NB003

NB003 (Previously AZD3229: Ningbo Newbay Technology Development Co., Ltd), was also designed as a broad inhibitor of *KIT* and *PDGFRA* mutant GIST. Preclinical in vitro testing demonstrated superior potency against both primary and secondary *KIT* mutations compared to imatinib and other approved agents.^99^ NB003 is currently undergoing phase 1 testing in patients with advanced GIST who progressed on or were intolerant to imatinib and other subsequent-line agents.^100^

### Manipulating Related Pathways

Preclinical models have shown that expression of ETV1, a member of the ETS family of transcription factors, is necessary for GIST tumor growth, and KIT activation promotes expression and stability of ETV1.^101,102^ Combined inhibition of KIT and ETV1 with imatinib and binimetinib (MEK-162), respectively, showed synergistic effect in in vitro and in vivo models.^103^ Phase I and II (NCT01991379) testing found this combination to be safe and well-tolerated in treatment-naïve patients.^104,105^ The ORR was 69%, and median PFS was 30 months.^105^

Heat-shock protein 90 (Hsp90), a chaperone molecule necessary for KIT and PDGFRA protein folding, emerged as a compelling target.^23^ Preclinical models of pimitespib (TAS-116), a novel Hsp90 inhibitor, showed efficacy in both imatinib-naïve and resistant cell lines.^106^ Phase I testing of pimitespib demonstrated safety with lower ocular and hepatotoxicity than prior Hsp90 inhibitors.^107^

CHAPTER-GIST-301 was a randomized, placebo-controlled phase III trial of pimitespib in 86 patients with multi-drug refractory GIST. Patients in the pimitespib arm had higher median PFS than placebo (2.8 vs. 1.4 months, HR 0.51, *P* = .0006) and were 58% less likely to die.^108^ A phase I trial of pimitespib in combination with imatinib (NCT05245968) is currently recruiting.^109^

mTOR inhibitors, such as everolimus, act downstream of mutant *KIT* and *PDGFRA* to reduce the activity of the PI3K/Akt/mTOR pathway. Preclinical testing demonstrated efficacy of combined mTOR and KIT inhibition in imatinib-resistant GIST.^110^ A phase I/II study of everolimus 2.5 mg daily and imatinib 600 mg daily, respectively, demonstrated an acceptable safety profile and potential efficacy with lengthened PFS after imatinib and sunitinib failure.^111^

### New Treatments for SDH-Deficient GIST

SDH-deficient GIST is characterized by epigenetic changes, in particular global DNA methylation, which leads to upregulation of fibroblast growth factor receptor (FGFR).^112-114^ This overexpression of FGFR has been implicated in the pathogenesis of SDH-deficient GIST, and FGFR inhibition in patient-derived, SDH-deficient xenograft mice models led to significant tumor reduction.^102^ Rogaratinib (BAY 1163877: Bayer), an oral pan-FGFR inhibitor (FRFR 1-4) is currently undergoing phase II testing in patients with FGFR1-4 altered sarcomas or SDH-deficient GIST (NCT04595747).^115^

## Conclusions

The management of advanced GIST has been revolutionized by improved understanding of molecular pathogenesis and emergence of oral targeted therapies. The majority (85%) of GIST are characterized by activating mutations in *KIT* or *PDGFRA*. Primary driver mutations can occur in the extracellular (*KIT* exon 9), juxta-membrane (*KIT* exon 11, *PDGFRA* exon 12), and ATP-binding site (*KIT* exon 13, *PDGFRA* exon 14) or activation loop (*KIT* exon 17, *PDGFRA* exon 18).

Imatinib was found to inhibit *KIT* and *PDGFRA* and became the first approved treatment for patients with GIST. Most patients benefited; however, treatment responses varied based on driver mutation with some, such as *PDGFRA* D842V and without *KIT* or *PGDFRA*, showing no response.

Nearly all patients eventually develop imatinib resistance as secondary mutations develop, typically in the ATP-binding domain or activation loop of the kinase domain. Sunitinib, regorafenib, and ripretinib were approved in response to imatinib resistance. Avapritinib was found to have impressive activity and approved as treatment in PDGFRA D842V mutations.

Future advances will hopefully come from the development of next generation KIT inhibitors, such as bezuclastinib (CGT9486), THE-630, and IDRX-42, and from inhibition of pathogenic processes, such as ETV1 (with binimetinib), Hsp90 (with pimitespib), and the PI3K/Akt/mTOR pathway. Finally, targeted treatments for SDH-deficient GIST, such as FGFR inhibition (with rogaratinib), are sorely needed.

## Data Availability

No new data were generated or analyzed in support of this research.

## References

[1] Ma GL , Murphy JD, Martinez ME, Sicklick JK. Epidemiology of gastrointestinal stromal tumors in the era of histology codes: results of a population-based study. Cancer Epidemiol Biomarkers Prev. 2015;24(1):298-302. 10.1158/1055-9965.EPI-14-1002. 25277795 PMC4294949

[2] Søreide K , Sandvik OM, Søreide JA, et al. Global epidemiology of gastrointestinal stromal tumours (GIST): a systematic review of population-based cohort studies. Cancer Epidemiol. 2016;40:39-46. 10.1016/j.canep.2015.10.031. 26618334

[3] Miettinen M , Sarlomo-Rikala M, Lasota J. Gastrointestinal stromal tumors: recent advances in understanding of their biology. Hum Pathol. 1999;30(10):1213-1220. 10.1016/s0046-8177(99)90040-0. 10534170

[4] Miettinen M , Lasota J. Gastrointestinal stromal tumors--definition, clinical, histological, immunohistochemical, and molecular genetic features and differential diagnosis. Virchows Arch. 2001;438(1):1-12. 10.1007/s004280000338. 11213830

[5] Benesch M , Wardelmann E, Ferrari A, Brennan B, Verschuur A. Gastrointestinal stromal tumors (GIST) in children and adolescents: a comprehensive review of the current literature. Pediatr Blood Cancer. 2009;53(7):1171-1179. 10.1002/pbc.22123. 19499582

[6] Joensuu H , Hohenberger P, Corless CL. Gastrointestinal stromal tumour. Lancet. 2013;382(9896):973-983. 10.1016/S0140-6736(13)60106-3. 23623056

[7] Miettinen M , Lasota J. Gastrointestinal stromal tumors: review on morphology, molecular pathology, prognosis, and differential diagnosis. Arch Pathol Lab Med. 2006;130(10):1466-1478. 10.5858/2006-130-1466-GSTROM. 17090188

[8] DeMatteo RP , Lewis JJ, Leung D, et al. Two hundred gastrointestinal stromal tumors: recurrence patterns and prognostic factors for survival. Ann Surg. 2000;231(1):51-58. 10.1097/00000658-200001000-00008. 10636102 PMC1420965

[9] Nickl NJ. Gastrointestinal stromal tumors: new progress, new questions. Curr Opin Gastroenterol. 2004;20(5):482-487. 10.1097/00001574-200409000-00011. 15689683

[10] vander Noot MR , Eloubeidi MA, Chen VK, et al. Diagnosis of gastrointestinal tract lesions by endoscopic ultrasound-guided fine-needle aspiration biopsy. Cancer. 2004;102(3):157-163. 10.1002/cncr.20360. 15211474

[11] Judson I , Demetri G. Advances in the treatment of gastrointestinal stromal tumours. Ann Oncol. 2007;18(Suppl 10):xx2020-xx2x24. 10.1093/annonc/mdm410. 17761719

[12] Joensuu H. Gastrointestinal stromal tumor (GIST). Ann Oncol. 2006;17(Suppl 10):x280-x286. 10.1093/annonc/mdl274. 17018739

[13] Novitsky YW , Kercher KW, Sing RF, Heniford BT. Long-term outcomes of laparoscopic resection of gastric gastrointestinal stromal tumors. Ann Surg. 2006;243(6):738-45; discussion 745. 10.1097/01.sla.0000219739.11758.27. 16772777 PMC1570564

[14] Chen K , Zhou YC, Mou YP, et al. Systematic review and meta-analysis of safety and efficacy of laparoscopic resection for gastrointestinal stromal tumors of the stomach. Surg Endosc. 2015;29(2):355-367. 10.1007/s00464-014-3676-6. 25005014

[15] Edmonson JH , Marks RS, Buckner JC, Mahoney MR. Contrast of response to dacarbazine, mitomycin, doxorubicin, and cisplatin (DMAP) plus GM-CSF between patients with advanced malignant gastrointestinal stromal tumors and patients with other advanced leiomyosarcomas. Cancer Invest. 2002;20(5-6):605-612. 10.1081/cnv-120002485. 12197215

[16] Rubin BP , Blanke CD, Demetri GD, et al.. Protocol for the examination of specimens from patients with gastrointestinal stromal tumor. Arch Pathol Lab Med. 2010;134(2):165-170. 10.5858/134.2.165. 20121601

[17] Sircar K , Hewlett BR, Huizinga JD, et al. Interstitial cells of Cajal as precursors of gastrointestinal stromal tumors. Am J Surg Pathol. 1999;23(4):377-389. 10.1097/00000478-199904000-00002. 10199467

[18] Wang L , Vargas H, French SW. Cellular origin of gastrointestinal stromal tumors: a study of 27 cases. Arch Pathol Lab Med. 2000;124(10):1471-1475. 10.5858/2000-124-1471-COOGST. 11035578

[19] Hirota S , Isozaki K, Moriyama Y, et al. Gain-of-function mutations of c-kit in human gastrointestinal stromal tumors. Science. 1998;279(5350):577-580. 10.1126/science.279.5350.577. 9438854

[20] Kindblom LG , Remotti HE, Aldenborg F, Meis-Kindblom JM. Gastrointestinal pacemaker cell tumor (GIPACT): gastrointestinal stromal tumors show phenotypic characteristics of the interstitial cells of Cajal. Am J Pathol. 1998;152(5):1259-1269. 9588894 PMC1858579

[21] Corless CL , Fletcher JA, Heinrich MC. Biology of gastrointestinal stromal tumors. J Clin Oncol. 2004;22(18):3813-3825. 10.1200/JCO.2004.05.140. 15365079

[22] Corless CL , Heinrich MC. Molecular pathobiology of gastrointestinal stromal sarcomas. Annu Rev Pathol. 2008;3:557-586. 10.1146/annurev.pathmechdis.3.121806.151538. 18039140

[23] Corless CL , Barnett CM, Heinrich MC. Gastrointestinal stromal tumours: origin and molecular oncology. Nat Rev Cancer. 2011;11(12):865-878. 10.1038/nrc3143. 22089421

[24] Heinrich MC , Owzar K, Corless CL, et al. Correlation of kinase genotype and clinical outcome in the North American Intergroup Phase III Trial of imatinib mesylate for treatment of advanced gastrointestinal stromal tumor: CALGB 150105 Study by Cancer and Leukemia Group B and Southwest Oncology Group. J Clin Oncol. 2008;26(33):5360-5367. 10.1200/JCO.2008.17.4284. 18955451 PMC2651078

[25] Gastrointestinal Stromal Tumor Meta-Analysis Group (MetaGIST). Comparison of two doses of imatinib for the treatment of unresectable or metastatic gastrointestinal stromal tumors: a meta-analysis of 1,640 patients. J Clin Oncol. 2010;28(7):1247-1253. 10.1200/JCO.2009.24.2099. 20124181 PMC2834472

[26] Gramza AW , Corless CL, Heinrich MC. Resistance to tyrosine kinase inhibitors in gastrointestinal stromal tumors. Clin Cancer Res. 2009;15(24):7510-7518. 10.1158/1078-0432.CCR-09-0190. 20008851

[27] Demetri GD , van Oosterom AT, Garrett CR, et al. Efficacy and safety of sunitinib in patients with advanced gastrointestinal stromal tumour after failure of imatinib: a randomised controlled trial. Lancet. 2006;368(9544):1329-1338. 10.1016/S0140-6736(06)69446-4. 17046465

[28] Heinrich MC , Maki RG, Corless CL, et al. Primary and secondary kinase genotypes correlate with the biological and clinical activity of sunitinib in imatinib-resistant gastrointestinal stromal tumor. J Clin Oncol. 2008;26(33):5352-5359. 10.1200/JCO.2007.15.7461. 18955458 PMC2651076

[29] Serrano C , Mariño-Enríquez A, Tao DL, et al. Complementary activity of tyrosine kinase inhibitors against secondary kit mutations in imatinib-resistant gastrointestinal stromal tumours. Br J Cancer. 2019;120(6):612-620. 10.1038/s41416-019-0389-6. 30792533 PMC6462042

[30] Smith BD , Kaufman MD, Lu WP, et al. Ripretinib (DCC-2618) is a switch control kinase inhibitor of a broad spectrum of oncogenic and drug-resistant KIT and PDGFRA variants. Cancer Cell. 2019;35(5):738-751.e9. 10.1016/j.ccell.2019.04.006. 31085175

[31] Evans EK , Gardino AK, Kim JL, et al. A precision therapy against cancers driven by KIT/PDGFRA mutations. Sci Transl Med. 2017;9(414):1-11. 10.1126/scitranslmed.aao1690. 29093181

[32] Heinrich MC , Jones RL, von Mehren M, et al. Avapritinib in advanced PDGFRA D842V-mutant gastrointestinal stromal tumour (NAVIGATOR): a multicentre, open-label, phase 1 trial. Lancet Oncol. 2020;21(7):935-946. 10.1016/S1470-2045(20)30269-2. 32615108

[33] Bauer S , Jones RL, Blay JY, et al. Ripretinib versus Sunitinib in patients with advanced gastrointestinal stromal tumor after treatment with Imatinib (INTRIGUE): a randomized, open-label, phase III trial. J Clin Oncol. 2022;40(34):3918-3928. 10.1200/JCO.22.00294. 35947817 PMC9746771

[34] Corless CL , Schroeder A, Griffith D, et al. PDGFRA mutations in gastrointestinal stromal tumors: frequency, spectrum and in vitro sensitivity to imatinib. J Clin Oncol. 2005;23(23):5357-5364. 10.1200/JCO.2005.14.068. 15928335

[35] Pauls K , Merkelbach-Bruse S, Thal D, Büttner R, Wardelmann E. PDGFRalpha- and c-kit-mutated gastrointestinal stromal tumours (GISTs) are characterized by distinctive histological and immunohistochemical features. Histopathology. 2005;46(2):166-175. 10.1111/j.1365-2559.2005.02061.x. 15693889

[36] Wardelmann E , Hrychyk A, Merkelbach-Bruse S, et al. Association of platelet-derived growth factor receptor alpha mutations with gastric primary site and epithelioid or mixed cell morphology in gastrointestinal stromal tumors. J Mol Diagn. 2004;6(3):197-204. 10.1016/s1525-1578(10)60510-7. 15269295 PMC1867629

[37] Boikos SA , Pappo AS, Killian JK, et al. Molecular subtypes of KIT/PDGFRA wild-type gastrointestinal stromal tumors: a report from the National Institutes of Health Gastrointestinal Stromal Tumor Clinic. JAMA Oncol. 2016;2(7):922-928. 10.1001/jamaoncol.2016.0256. 27011036 PMC5472100

[38] Carney JA. Gastric stromal sarcoma, pulmonary chondroma, and extra-adrenal paraganglioma (Carney Triad): natural history, adrenocortical component, and possible familial occurrence. Mayo Clin Proc. 1999;74(6):543-552. 10.4065/74.6.543. 10377927

[39] Agaimy A , Pelz AF, Corless CL, et al. Epithelioid gastric stromal tumours of the antrum in young females with the Carney triad: a report of three new cases with mutational analysis and comparative genomic hybridization. Oncol Rep. 2007;18(1):9-15. 17549339

[40] Hostein I , Faur N, Primois C, et al. BRAF mutation status in gastrointestinal stromal tumors. Am J Clin Pathol. 2010;133(1):141-148. 10.1309/AJCPPCKGA2QGBJ1R. 20023270

[41] Agaram NP , Wong GC, Guo T, et al. Novel V600E BRAF mutations in imatinib-naive and imatinib-resistant gastrointestinal stromal tumors. Genes Chromosomes Cancer. 2008;47(10):853-859. 10.1002/gcc.20589. 18615679 PMC2902874

[42] Atiq MA , Davis JL, Hornick JL, et al. Mesenchymal tumors of the gastrointestinal tract with NTRK rearrangements: a clinicopathological, immunophenotypic, and molecular study of eight cases, emphasizing their distinction from gastrointestinal stromal tumor (GIST). Mod Pathol. 2021;34(1):95-103. 10.1038/s41379-020-0623-z. 32669612

[43] Cohen MH , Williams G, Johnson JR, et al. Approval summary for imatinib mesylate capsules in the treatment of chronic myelogenous leukemia. Clin Cancer Res. 2002;8(5):935-942. 12006504

[44] Demetri GD , von Mehren M, Blanke CD, et al. Efficacy and safety of imatinib mesylate in advanced gastrointestinal stromal tumors. N Engl J Med. 2002;347(7):472-480. 10.1056/NEJMoa020461. 12181401

[45] Blanke CD , Rankin C, Demetri GD, et al. Phase III randomized, intergroup trial assessing imatinib mesylate at two dose levels in patients with unresectable or metastatic gastrointestinal stromal tumors expressing the kit receptor tyrosine kinase: S0033. J Clin Oncol. 2008;26(4):626-632. 10.1200/JCO.2007.13.4452. 18235122

[46] Dagher R , Cohen M, Williams G, et al. Approval summary: imatinib mesylate in the treatment of metastatic and/or unresectable malignant gastrointestinal stromal tumors. Clin Cancer Res. 2002;8(10):3034-3038. 12374669

[47] Heinrich MC , Corless CL, Demetri GD, et al. Kinase mutations and imatinib response in patients with metastatic gastrointestinal stromal tumor. J Clin Oncol. 2003;21(23):4342-4349. 10.1200/JCO.2003.04.190. 14645423

[48] Antonescu CR , Besmer P, Guo T, et al. Acquired resistance to imatinib in gastrointestinal stromal tumor occurs through secondary gene mutation. Clin Cancer Res. 2005;11(11):4182-4190. 10.1158/1078-0432.CCR-04-2245. 15930355

[49] Chen LL , Trent JC, Wu EF, et al. A missense mutation in KIT kinase domain 1 correlates with imatinib resistance in gastrointestinal stromal tumors. Cancer Res. 2004;64(17):5913-5919. 10.1158/0008-5472.CAN-04-0085. 15342366

[50] Debiec-Rychter M , Cools J, Dumez H, et al. Mechanisms of resistance to imatinib mesylate in gastrointestinal stromal tumors and activity of the PKC412 inhibitor against imatinib-resistant mutants. Gastroenterology. 2005;128(2):270-279. 10.1053/j.gastro.2004.11.020. 15685537

[51] Liegl B , Kepten I, Le C, et al. Heterogeneity of kinase inhibitor resistance mechanisms in GIST. J Pathol. 2008;216(1):64-74. 10.1002/path.2382. 18623623 PMC2693040

[52] Reichardt P , Kang YK, Rutkowski P, et al. Clinical outcomes of patients with advanced gastrointestinal stromal tumors: safety and efficacy in a worldwide treatment-use trial of sunitinib. Cancer. 2015;121(9):1405-1413. 10.1002/cncr.29220. 25641662 PMC4442000

[53] Demetri GD , Garrett CR, Schöffski P, et al. Complete longitudinal analyses of the randomized, placebo-controlled, phase III trial of sunitinib in patients with gastrointestinal stromal tumor following imatinib failure. Clin Cancer Res. 2012;18(11):3170-3179. 10.1158/1078-0432.CCR-11-3005. 22661587 PMC4030710

[54] Goodman VL , Rock EP, Dagher R, et al. Approval summary: sunitinib for the treatment of imatinib refractory or intolerant gastrointestinal stromal tumors and advanced renal cell carcinoma. Clin Cancer Res. 2007;13(5):1367-1373. 10.1158/1078-0432.CCR-06-2328. 17332278

[55] George S , Wang Q, Heinrich MC, et al. Efficacy and safety of regorafenib in patients with metastatic and/or unresectable GI stromal tumor after failure of imatinib and sunitinib: a multicenter phase II trial. J Clin Oncol. 2012;30(19):2401-2407. 10.1200/JCO.2011.39.9394. 22614970 PMC3675695

[56] Demetri GD , Reichardt P, Kang YK, et al.. Efficacy and safety of regorafenib for advanced gastrointestinal stromal tumours after failure of imatinib and sunitinib (GRID): an international, multicentre, randomised, placebo-controlled, phase 3 trial. Lancet. 2013;381(9863):295-302. 10.1016/S0140-6736(12)61857-1. 23177515 PMC3819942

[57] Food and Drug Administration (FDA). FDA approves avapritinib for gastrointestinal stromal tumor with a rare mutation. Published January 9, 2020. Accessed December 21, 2022. https://www.fda.gov/drugs/resources-information-approved-drugs/fda-approves-avapritinib-gastrointestinal-stromal-tumor-rare-mutation

[58] Trullas-Jimeno A , Delgado J, Garcia-Ochoa B, et al. The EMA assessment of avapritinib in the treatment of gastrointestinal stromal tumours harbouring the PDGFRA D842V mutation. ESMO Open. 2021;6(3):100159100159. 10.1016/j.esmoop.2021.100159. PMC8165402 34023541

[59] Kumar V , Doros L, Thompson M, et al. FDA approval summary: ripretinib for advanced gastrointestinal stromal tumor. Clin Cancer Res. Published online December 9, 2022;29(11):2022-2024. 10.1158/1078-0432.CCR-22-2400. PMC10238554 36485007

[60] Klug LR , Kent JD, Heinrich MC. Structural and clinical consequences of activation loop mutations in class III receptor tyrosine kinases. Pharmacol Ther. 2018;191:123-134. 10.1016/j.pharmthera.2018.06.016. 29964125

[61] Indio V , Astolfi A, Tarantino G, et al. Integrated molecular characterization of gastrointestinal stromal tumors (GIST) harboring the rare D842V mutation in PDGFRA gene. Int J Mol Sci. 2018;19(3):732-732. 10.3390/ijms19030732. 29510530 PMC5877593

[62] Jones RL , Serrano C, von Mehren M, et al. Avapritinib in unresectable or metastatic PDGFRA D842V-mutant gastrointestinal stromal tumours: long-term efficacy and safety data from the NAVIGATOR phase I trial. Eur J Cancer. 2021;145(2021):132-142. 10.1016/j.ejca.2020.12.008. 33465704 PMC9518931

[63] Janeway KA , Albritton KH, Van Den Abbeele AD, et al. Sunitinib treatment in pediatric patients with advanced GIST following failure of imatinib. Pediatr Blood Cancer. 2009;52(7):767-771. 10.1002/pbc.21909. 19326424

[64] Mir O , Cropet C, Toulmonde M, et al.. Pazopanib plus best supportive care versus best supportive care alone in advanced gastrointestinal stromal tumours resistant to imatinib and sunitinib (PAZOGIST): a randomised, multicentre, open-label phase 2 trial. Lancet Oncol. 2016;17(5):632-641. 10.1016/S1470-2045(16)00075-9. 27068858

[65] Joensuu H. Risk stratification of patients diagnosed with gastrointestinal stromal tumor. Hum Pathol. 2008;39(10):1411-1419. 10.1016/j.humpath.2008.06.025. 18774375

[66] Dematteo RP , Ballman KV, Antonescu CR, et al; American College of Surgeons Oncology Group (ACOSOG) Intergroup Adjuvant GIST Study Team. Adjuvant imatinib mesylate after resection of localised, primary gastrointestinal stromal tumour: a randomised, double-blind, placebo-controlled trial. Lancet. 2009;373(9669):1097-1104. 10.1016/S0140-6736(09)60500-6. 19303137 PMC2915459

[67] Casali PG , le Cesne A, Poveda Velasco A, et al. Time to definitive failure to the first tyrosine kinase inhibitor in localized GI stromal tumors treated with imatinib as an adjuvant: a European organisation for research and treatment of cancer soft tissue and bone sarcoma group intergroup randomized trial in collaboration with the Australasian gastro-intestinal trials group, UNICANCER, French sarcoma group, Italian sarcoma group, and Spanish group for research on sarcomas. J Clin Oncol. 2015;33(36):4276-4283. 10.1200/JCO.2015.62.4304. 26573069

[68] Casali PG , le Cesne A, Velasco AP, et al. Final analysis of the randomized trial on imatinib as an adjuvant in localized gastrointestinal stromal tumors (GIST) from the eortc soft tissue and bone sarcoma group (STBSG), the Australasian gastro-intestinal trials group (AGITG), UNICANCER, French sarcoma group (FSG), Italian sarcoma group (ISG), and Spanish group for research on sarcomas (GEIS)☆. Ann Oncol. 2021;32(4):533-541. 10.1016/j.annonc.2021.01.004. 33482247

[69] Joensuu H , Eriksson M, Sundby Hall K, et al. One vs three years of adjuvant imatinib for operable gastrointestinal stromal tumor: a randomized trial. JAMA. 2012;307(12):1265-1272. 10.1001/jama.2012.347. 22453568

[70] Joensuu H , Wardelmann E, Sihto H, et al. Effect of KIT and PDGFRA mutations on survival in patients with gastrointestinal stromal tumors treated with adjuvant imatinib: an exploratory analysis of a randomized clinical trial. JAMA Oncol. 2017;3(5):602-609. 10.1001/jamaoncol.2016.5751. 28334365 PMC5470395

[71] Raut CP , Espat NJ, Maki RG, et al. Efficacy and tolerability of 5-year adjuvant imatinib treatment for patients with resected intermediate- or high-risk primary gastrointestinal stromal tumor: the PERSIST-5 clinical trial. JAMA Oncol. 2018;4(12):e184060e184060. 10.1001/jamaoncol.2018.4060. 30383140 PMC6440723

[72] Lin JX , Chen QF, Zheng CH, et al. Is 3-years duration of adjuvant imatinib mesylate treatment sufficient for patients with high-risk gastrointestinal stromal tumor? A study based on long-term follow-up. J Cancer Res Clin Oncol. 2017;143(4):727-734. 10.1007/s00432-016-2334-x. 28083710 PMC11819384

[73] Blanke CD , DeMatteo RP. Duration of adjuvant therapy for patients with gastrointestinal stromal tumors: where is goldilocks when we need her? JAMA Oncol. 2016;2(6):721-722. 10.1001/jamaoncol.2016.0094. 27031092

[74] Joensuu H , Eriksson M, Sundby Hall K, et al. Adjuvant imatinib for high-risk GI stromal tumor: analysis of a randomized trial. J Clin Oncol. 2016;34(3):244-250. 10.1200/JCO.2015.62.9170. 26527782

[75] Zalcberg JR , Verweij J, Casali PG, et al. Outcome of patients with advanced gastro-intestinal stromal tumours crossing over to a daily imatinib dose of 800 mg after progression on 400 mg. Eur J Cancer. 2005;41(12):1751-1757. 10.1016/j.ejca.2005.04.034. 16098458

[76] Yoo C , Ryu MH, Jo J, et al. Efficacy of imatinib in patients with platelet-derived growth factor receptor alpha-mutated gastrointestinal stromal tumors. Cancer Res Treat. 2016;48(2):546-552. 10.4143/crt.2015.015. 26130666 PMC4843750

[77] Cassier PA , Fumagalli E, Rutkowski P, et al; European Organisation for Research and Treatment of Cancer. Outcome of patients with platelet-derived growth factor receptor alpha-mutated gastrointestinal stromal tumors in the tyrosine kinase inhibitor era. Clin Cancer Res. 2012;18(16):4458-4464. 10.1158/1078-0432.CCR-11-3025. 22718859

[78] George S , Merriam P, Maki RG, et al. Multicenter phase II trial of sunitinib in the treatment of nongastrointestinal stromal tumor sarcomas. J Clin Oncol. 2009;27(19):3154-3160. 10.1200/JCO.2008.20.9890. 19451429 PMC2716937

[79] Blay JY , Serrano C, Heinrich MC, et al. Ripretinib in patients with advanced gastrointestinal stromal tumours (INVICTUS): a double-blind, randomised, placebo-controlled, phase 3 trial. Lancet Oncol. 2020;21(7):923-934. 10.1016/S1470-2045(20)30168-6. 32511981 PMC8383051

[80] George S , Chi P, Heinrich MC, et al. Ripretinib intrapatient dose escalation after disease progression provides clinically meaningful outcomes in advanced gastrointestinal stromal tumour. Eur J Cancer. 2021;155(2021):236-244. 10.1016/j.ejca.2021.07.010. 34391056 PMC9362852

[81] Zalcberg JR , Heinrich MC, George S, et al. Clinical benefit of ripretinib dose escalation after disease progression in advanced gastrointestinal stromal tumor: an analysis of the INVICTUS study. Oncologist. 2021;26(11):e2053-e2060. 10.1002/onco.13917. 34313371 PMC8571742

[82] Bauer S , Jones RL, George S, et al. Mutational heterogeneity of imatinib resistance and efficacy of ripretinib vs sunitinib in patients with gastrointestinal stromal tumor: ctDNA analysis from INTRIGUE. J Clin Oncol. 2023;41(36_suppl):397784-397784. 10.1200/jco.2023.41.36_suppl.397784.

[83] National Comprehensive Cancer Network (NCCN) Clinical Practice Guidelines in Oncology (NCCN Guidelines). Gastrointestinal Stromal Tumors. Published March 13, 2023. Accessed April 18, 2023. https://www.nccn.org/professionals/physician_gls/pdf/gist.pdf

[84] Clinicaltrials.gov. A Study of Ripretinib vs Sunitinib in Patients With Advanced GIST With Specific KIT Exon Mutations Who Were Previously Treated With Imatinib (INSIGHT): NCT05734105. National Library of Medicine. Published 2023. Accessed April 23, 2023. https://clinicaltrials.gov/ct2/show/NCT05734105

[85] Kang YK , George S, Jones RL, et al. Avapritinib versus regorafenib in locally advanced unresectable or metastatic GI stromal tumor: a randomized, open-label phase III study. J Clin Oncol. 2021;39(28):3128-3139. 10.1200/JCO.21.00217. 34343033 PMC8478403

[86] Johansson G , Berndsen M, Lindskog S, et al. Monitoring circulating tumor DNA during surgical treatment in patients with gastrointestinal stromal tumors. Mol Cancer Ther. 2021;20(12):2568-2576. 10.1158/1535-7163.MCT-21-0403. 34552011 PMC9398151

[87] Ko TK , Lee E, Ng CCY, et al. Circulating tumor DNA mutations in progressive gastrointestinal stromal tumors identify biomarkers of treatment resistance and uncover potential therapeutic strategies. Front Oncol. 2022;12(2022):840843. 10.3389/fonc.2022.840843. 35273917 PMC8904145

[88] Jilg S , Rassner M, Maier J, et al. Circulating cKIT and PDGFRA DNA indicates disease activity in gastrointestinal stromal tumor (GIST). Int J Cancer. 2019;145(8):2292-2303. 10.1002/ijc.32282. 30882891

[89] Serrano C , Leal A, Kuang Y, et al. Phase I study of rapid alternation of sunitinib and regorafenib for the treatment of tyrosine kinase inhibitor refractory gastrointestinal stromal tumors. Clin Cancer Res. 2019;25(24):7287-7293. 10.1158/1078-0432.CCR-19-2150. 31471313

[90] Serrano C , Bauer S, Gómez-Peregrina D, et al. Circulating tumor DNA (ctDNA) analyses of the phase III VOYAGER trial: KIT mutational landscape and outcomes in patients with advanced gastrointestinal stromal tumor (GIST). J Clin Oncol. 2022;40(16_suppl):101-101. 10.1200/jco.2022.40.16_suppl.101. PMC10330293 37105265

[91] Arshad J , Roberts A, Ahmed J, et al. Utility of circulating tumor DNA in the management of patients with GI stromal tumor: analysis of 243 patients. JCO Precis Oncol. 2020;(4):66-73. 10.1200/PO.19.00253. 35050730

[92] Gebreyohannes YK , Burton EA, Wozniak A, et al. PLX9486 shows anti-tumor efficacy in patient-derived, tyrosine kinase inhibitor-resistant KIT-mutant xenograft models of gastrointestinal stromal tumors. Clin Exp Med. 2019;19(2):201-210. 10.1007/s10238-018-0541-2. 30523507

[93] Wagner AJ , Severson PL, Shields AF, et al. Association of combination of conformation-specific KIT inhibitors with clinical benefit in patients with refractory gastrointestinal stromal tumors: a phase 1b/2a nonrandomized clinical trial. JAMA Oncol. 2021;7(9):1343-1350. 10.1001/jamaoncol.2021.2086. 34236401 PMC8267845

[94] Clinicaltrials.gov. (Peak) A Phase 3 Randomized Trial of CGT9486+Sunitinib vs. Sunitinib in Subjects With Gastrointestinal Stromal Tumors: NCT05208047. National Library of Medicine (US). Published January 26, 2022. Accessed December 24, 2022. https://clinicaltrials.gov/ct2/show/NCT05208047

[95] de Sutter L , Wozniak A, Verreet J, et al. Abstract 2666: anti-tumor effects of the novel *KIT* mutant inhibitor M4205 in patient-derived gastrointestinal stromal tumor (GIST) xenograft models. Cancer Res. 2022;82(12_Supplement):2666-2666. 10.1158/1538-7445.am2022-2666.

[96] Clinicaltrials.gov. A First-in-human (FIH) Study of IDRX-42 in Participants With Metastatic and/or Unresectable Gastrointestinal Stromal Tumors: NCT05489237. National Library of Medicine (US). Published August 5, 2022. Accessed December 24, 2022. https://clinicaltrials.gov/ct2/show/NCT05489237

[97] Rivera VM , Huang WS, Lu M, et al. Abstract 1292: preclinical characterization of THE-630, a next-generation inhibitor for KIT-mutant gastrointestinal stromal tumors (GIST). Cancer Res. 2021;81(13_Supplement):1292-1292. 10.1158/1538-7445.am2021-1292.

[98] Clinicaltrials.gov. A Study of THE-630 in Patients With Advanced Gastrointestinal Stromal Tumors (GIST): NCT05160168. National Library of Medicine (US). Published December 16, 2021. Accessed December 24, 2022. https://clinicaltrials.gov/ct2/show/NCT05160168

[99] Banks E , Grondine M, Bhavsar D, et al. Discovery and pharmacological characterization of AZD3229, a potent KIT/PDGFRα inhibitor for treatment of gastrointestinal stromal tumors. Sci Transl Med. 2020;12(541):1-13. 10.1126/scitranslmed.aaz2481. 32350132

[100] Clinicaltrials.gov. A Study of NB003 in Patients With Advanced Malignancies: NCT04936178. National Library of Medicine. Published 2021. Accessed April 18, 2023. https://clinicaltrials.gov/ct2/show/NCT04936178

[101] Rosenbaum E , Kelly C, D’Angelo SP, et al. A phase I study of binimetinib (MEK162) combined with pexidartinib (PLX3397) in patients with advanced gastrointestinal stromal tumor. Oncologist. 2019;24(10):1309-e983. 10.1634/theoncologist.2019-0418. 31213500 PMC6795162

[102] Chi P , Chen Y, Zhang L, et al. ETV1 is a lineage survival factor that cooperates with KIT in gastrointestinal stromal tumours. Nature. 2010;467(7317):849-853. 10.1038/nature09409. 20927104 PMC2955195

[103] Ran L , Sirota I, Cao Z, et al. Combined inhibition of MAP kinase and KIT signaling synergistically destabilizes ETV1 and suppresses GIST tumor growth. Cancer Discov. 2015;5(3):304-315. 10.1158/2159-8290.CD-14-0985. 25572173 PMC4355391

[104] Chi P , Qin LX, Camacho N, et al. Phase Ib trial of the combination of imatinib and binimetinib in patients with advanced gastrointestinal stromal tumors. Clin Cancer Res. 2022;28(8):1507-1517. 10.1158/1078-0432.CCR-21-3909. 35110417 PMC9012681

[105] Chi P , Qin LX, Nguyen B, et al. Phase II trial of imatinib plus binimetinib in patients with treatment-naive advanced gastrointestinal stromal tumor. J Clin Oncol. 2022;40(9):997-1008. 10.1200/JCO.21.02029. 35041493 PMC8937014

[106] Saito Y , Takahashi T, Obata Y, et al. TAS-116 inhibits oncogenic KIT signalling on the Golgi in both imatinib-naïve and imatinib-resistant gastrointestinal stromal tumours. Br J Cancer. 2020;122(5):658-667. 10.1038/s41416-019-0688-y. 31857719 PMC7054534

[107] Shimomura A , Yamamoto N, Kondo S, et al. First-in-human phase I study of an oral HSP90 inhibitor, TAS-116, in patients with advanced solid tumors. Mol Cancer Ther. 2019;18(3):531-540. 10.1158/1535-7163.MCT-18-0831. 30679388

[108] Kurokawa Y , Honma Y, Sawaki A, et al. Pimitespib in patients with advanced gastrointestinal stromal tumor (CHAPTER-GIST-301): a randomized, double-blind, placebo-controlled phase III trial. Ann Oncol. 2022;33(9):959-967. 10.1016/j.annonc.2022.05.518. 35688358

[109] Clinicaltrials.gov. A study of pimitespib in combination with imatinib in patients with GIST (CHAPTER-GIST-101): NCT05245968. National Library of Medicine (US). Published February 18, 2022. Accessed December 24, 2022. https://clinicaltrials.gov/ct2/show/NCT05245968

[110] Bauer S , Duensing A, Demetri GD, Fletcher JA. KIT oncogenic signaling mechanisms in imatinib-resistant gastrointestinal stromal tumor: PI3-kinase/AKT is a crucial survival pathway. Oncogene. 2007;26(54):7560-7568. 10.1038/sj.onc.1210558. 17546049

[111] Schöffski P , Reichardt P, Blay JY, et al. A phase I-II study of everolimus (RAD001) in combination with imatinib in patients with imatinib-resistant gastrointestinal stromal tumors. Ann Oncol. 2010;21(10):1990-1998. 10.1093/annonc/mdq076. 20507881

[112] Astolfi A , Pantaleo MA, Indio V, Urbini M, Nannini M. The emerging role of the FGF/FGFR pathway in gastrointestinal stromal tumor. Int J Mol Sci. 2020;21(9):3313-3313. 10.3390/ijms21093313. 32392832 PMC7246647

[113] Nannini M , Rizzo A, Indio V, et al. Targeted therapy in SDH-deficient GIST. Ther Adv Med Oncol. 2021;13(2021):17588359211023278. 10.1177/17588359211023278. 34262616 PMC8246492

[114] Flavahan WA , Drier Y, Johnstone SE, et al. Altered chromosomal topology drives oncogenic programs in SDH-deficient GISTs. Nature. 2019;575(7781):229-233. 10.1038/s41586-019-1668-3. 31666694 PMC6913936

[115] Clinicaltrials.gov. Phase 2 Study of Rogaratinib (BAY 1163877) in the Treatment of Patients With Sarcoma Harboring Alterations in Fibroblast Growth Factor Receptor (FGFR) 1-4 and SDH-Deficient Gastrointestinal Stromal Tumor (GIST): NCT04595747. National Library of Medicine (US). Published October 22, 2020. Accessed December 24, 2022. https://clinicaltrials.gov/ct2/show/NCT04595747

